# Supplementary material for: Role of Cr Element in Highly Dense Passivation of Fe-Based Amorphous Alloy
Source: Materials (Basel). 2023 Oct 10;16(20):6630. doi: 10.3390/ma16206630 (PMC10608733; doi:10.3390/ma16206630)
Supplement: Supplementary file 1 [file materials-16-06630-s001.zip › materials-2630464-supplementary.pdf]

materials

Role of Cr Element in Highly Dense Passivation of Fe-Based Amorphous Alloy

Ziqi Song [1], Zhaoxuan Wang [1], Qi Chen [1], Zhigang Qi [1], Ki Buem Kim [2] and Weimin Wang [1,*]

[1] Key Laboratory for Liquid-Solid Structural Evolution and Processing of Materials (Ministry of Education), School of Materials Science and Engineering, Shandong University, Jinan 250061, China; 202234218@mail.sdu.edu.cn (Z.S.); 202114121@mail.sdu.edu.cn (Z.W.); 202120538@mail.sdu.edu.cn (Q.C.); 202034203@mail.sdu.edu.cn (Z.Q.)

[2] Department of Nanotechnology and Advanced Materials Engineering, Sejong University, Seoul 05006, Republic of Korea; kbkim@sejong.ac.kr

* Correspondence: weiminw@sdu.edu.cn

1. The Supplementary Figures and Tables

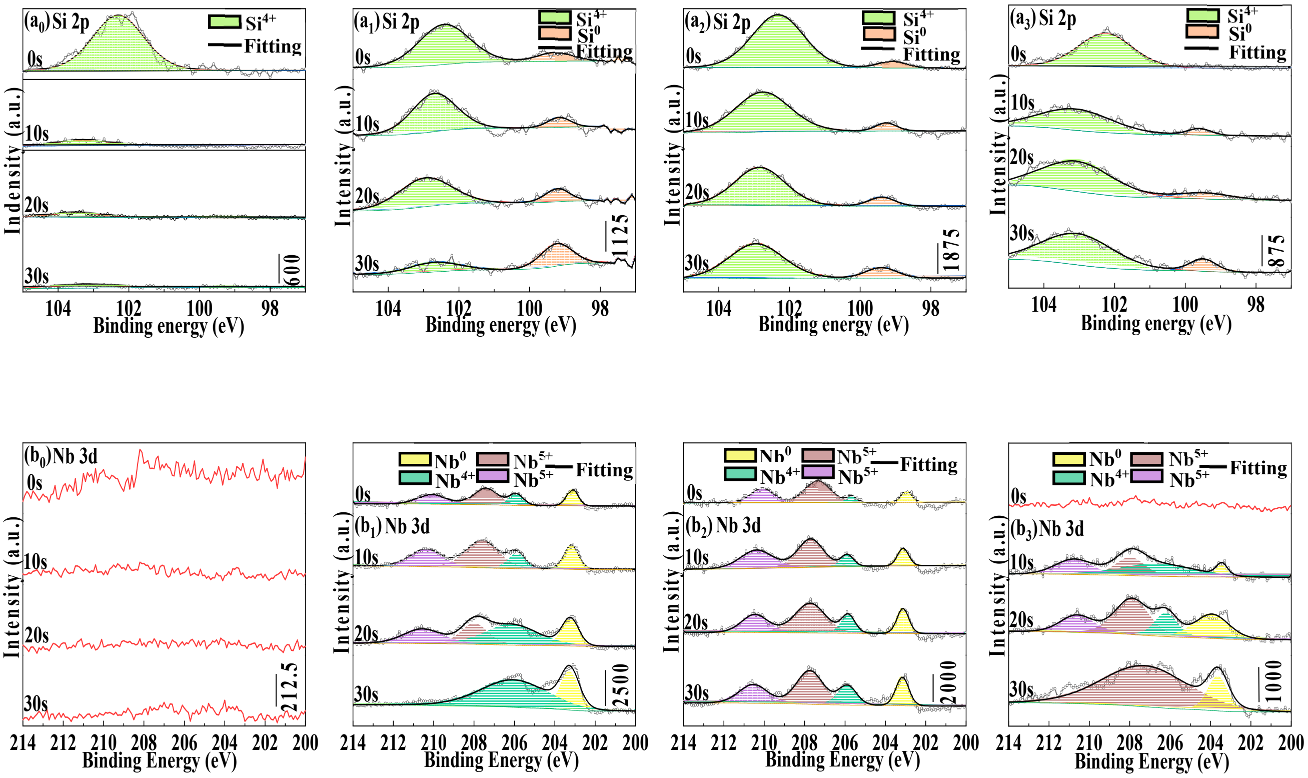

Figure S1. XPS spectra of Si 2p and Nb 3d in binding energy regions for the as-spun $Fe_{72-x}Cr_xB_{19.2}Si_{4.8}Nb_4$ ribbons with $x = 0$ (Cr0), $x = 7.2$ (Cr7), $x = 21.6$ (Cr21), and $x = 36$ (Cr36) after corrosion in neutral solutions.

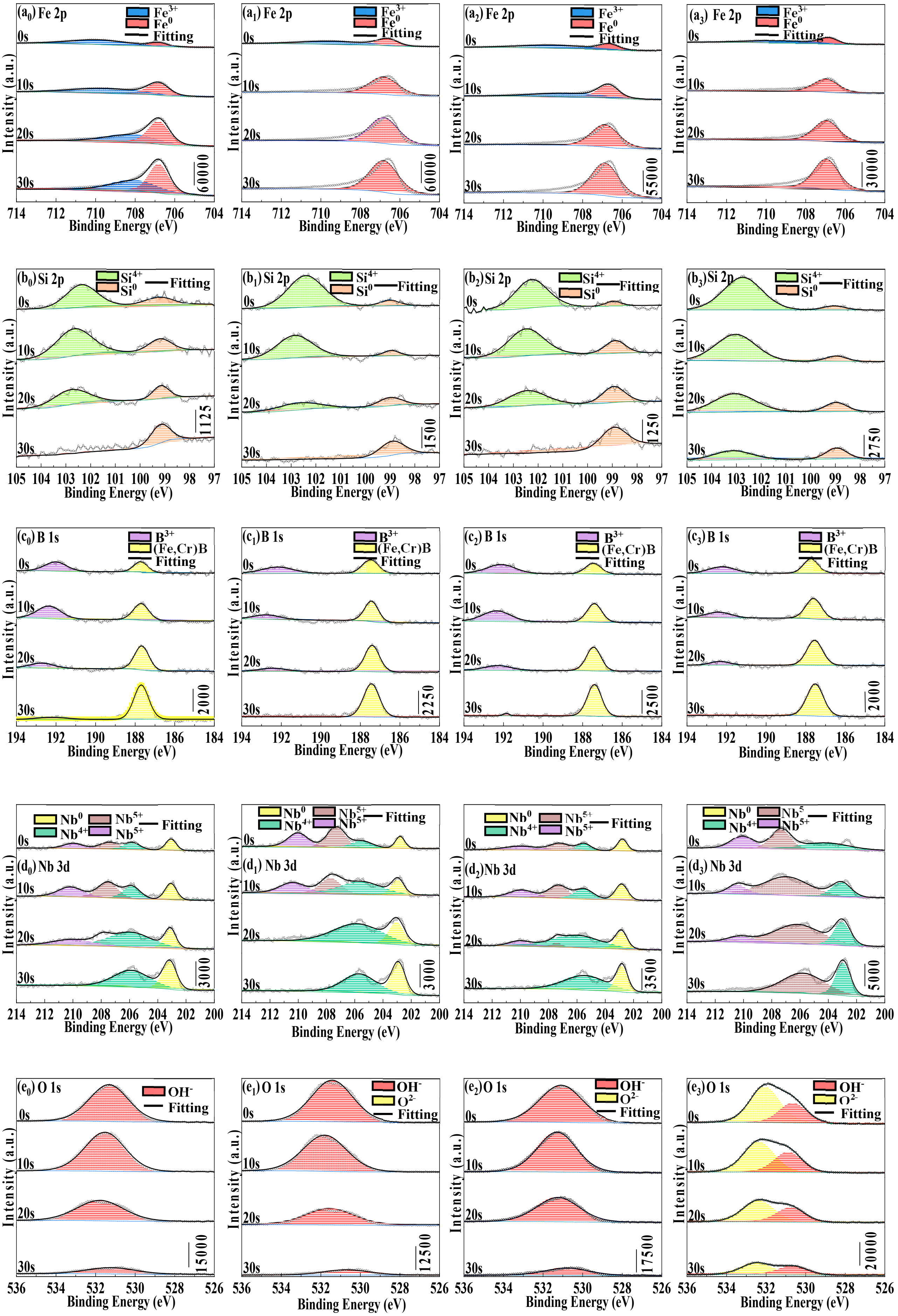

(a0) Fe 2p
(a1) Fe 2p
(a2) Fe 2p
(a3) Fe 2p
Fe3+
Fe0
Fitting
0s
10s
20s
30s
60000
55000
30000
(b0) Si 2p
(b1) Si 2p
(b2) Si 2p
(b3) Si 2p
Si4+
Si0
Fitting
1125
1500
1250
2750
(c0) B 1s
(c1) B 1s
(c2) B 1s
(c3) B 1s
B3+
(Fe,Cr)B
Fitting
2000
2250
2500
(d0) Nb 3d
(d1) Nb 3d
(d2) Nb 3d
(d3) Nb 3d
Nb0
Nb4+
Nb5+
Fitting
3000
3500
5000
(e0) O 1s
(e1) O 1s
(e2) O 1s
(e3) O 1s
OH-
O2-
Fitting
15000
12500
17500
20000
Intensity (a.u.)
Binding Energy (eV)

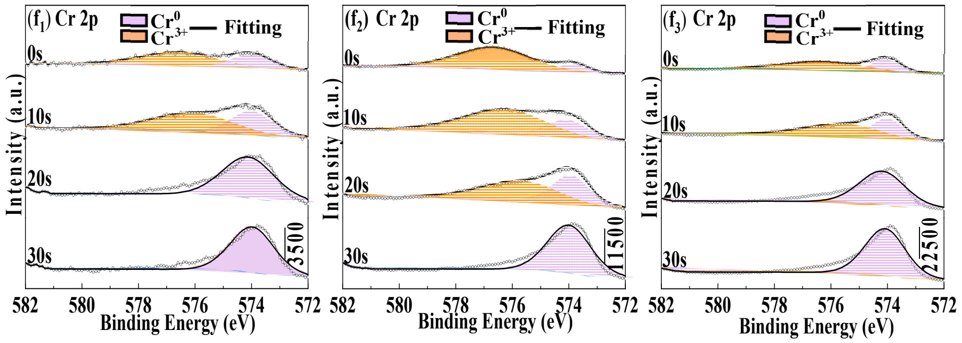

Figure S2. XPS spectra of Fe 2p, Si 2p, B 1s, Nb 3d O 1s and Cr 2p in binding energy regions for the as-spun $Fe_{72-x}Cr_xB_{19.2}Si_{4.8}Nb_4$ ribbons with x = 0 (Cr0), x = 7.2 (Cr7), x = 21.6 (Cr21), and x = 36 (Cr36).

Table S1. The comparison data of corrosion potential (E_{corr}) and corrosion current density (i_{corr}) of various Fe-based alloys in 3.5 wt.% NaCl solution.

Sample	E_{corr} (V)	i_{corr} (μA cm^{-2})	References
Cr0	-0.69	40.72	
Cr7	-0.56	14.83	
Cr21	-0.55	8.43	
Cr36	-0.50	5.28	
$Fe_{66}B_{30}Nb_4$	-0.7	15	[1]
$[(Fe_{0.6}Co_{0.4})_{0.75}B_{0.2}Si_{0.05}]_{96}Nb_4$	-0.55	2	
$[(Fe_{0.7}Co_{0.3})_{0.75}B_{0.2}Si_{0.05}]_{96}Nb_4$	-0.63	7	
$Fe_{56}Cr_{23}Ni_{5.7}B_{16}$	-0.13	0.015	
$Fe_{53}Cr_{22}Ni_{5.6}B_{19}$	-0.2	0.06	
$Fe_{50}Cr_{22}Ni_{5.4}B_{23}$	-0.17	0.02	
SS 316LN	-0.2	0.2	
$Fe_{82}B_{10}P_4Si_3C$	-0.64	41.7	[2]
$Fe_{82}Cr_2B_8P_4Si_3C$	-0.63	15.3	
$Fe_{82}NbB_9P_4Si_3C$	-0.49	18.2	
$Fe_{82}Cr_2NbB_8P_4Si_2C$	-0.42	4.06	
$Fe_{84}Mo_2B_8Si_2P_4$	-0.68	6.5	[3]
$Fe_{83}Mo_2B_8Si_2P_4C_1$	-0.68	1.54	
$Fe_{84}Cr_2B_8Si_2P_4$	-0.69	0.68	
$Fe_{83}Cr_2B_8Si_2P_4C_1$	-0.67	2.05	
FeCrMoNiB	-0.262	1.89	
$Fe_{83.3}B_{16}Cu_{0.7}$	-0.743	30	[4]
$Fe_{83.3}Si_1B_{15}Cu_{0.7}$	-0.738	16	
$Fe_{83.3}Si_2B_{14}Cu_{0.7}$	-0.737	22	
$Fe_{83.3}Si_3B_{13}Cu_{0.7}$	-0.736	29	
$Fe_{72}Ni_8Si_{10}B_{10}$	-0.501	15.4	[5]
FeNiB	-0.522	13	[6]
FeNiB+6%Cr	-0.394	4.4	
FeNiB+11% Cr	-0.272	0.29	
FeNiB+17% Cr	-0.256	0.15	
FeNiB+26% Cr	-0.264	0.15	
FeNiB+33% Cr	-0.256	0.14	
$Fe_{48}Cr_{23}Mo_{10}C_{14}B_5$	-0.859	3.03	[7]
$Fe_{68}Cr_8Mo_4Nb_4B_{16}$	-0.145	0.75	[8]
$Fe_{60}Cr_8Nb_8B_{24}$	-0.23	0.41	[9]
$Fe_{75.8}Si_{12}B_8Nb_{2.6}Cu_{0.6}P_1$	-0.579	17.4	[10]
$Fe_{38}Co_{38}Mo_8Cu_1B_{15}$	-0.597	8.21	[11]
$Fe_{39.5}Co_{39.5}Mo_8Cu_1B_{12}$	-0.691	20.2	
$Fe_{42}Co_{42}Cu_1B_{15}$	-0.658	12.9	
$Fe_{63}Co_{21}Cu_1B_{15}$	-0.723	33.1	
$Fe_{84}Cu_1B_{15}$	-0.874	36	
$Fe_{85}B_{15}$	-1.010	64.7	
Fe-Cr-Mo-C-B	-0.799	145	[12]
Fe-Cr-P-B-C	-0.524	3.2	

Fe-Cr-Mo-Ni-B-Co-Cu-Si-C	-0.56	3.92
Fe-Cr-Mo-Ni-P-B-C-Si	-0.45	8.3
Fe-Cr-Mo-C-B-Y	-0.559	4.12
Fe-Cr-Mo-C-B	-0.546	10
Fe-C-Si-B-P-Cr-Mo-Al	-0.707	2.3
Fe-Cr-Mo-C-B-Y	-0.458	11.3
Fe-Cr-Si-B-C	-0.641	31.85
Fe-Cr-P-B-C	-0.647	4.3
Fe-Cr-Mo-C-B-Y	-0.679	5.091
Fe-Mo-Cr-Co	-0.438	6.9
Fe-Cr-Mo-B-C	-0.333	52.2
Fe-Cr-Mo-B-C-P	-0.415	1.1

The Supplementary References:

1. Botta, W. J.; Berger, J. E.; Kiminami, C. S.; Roche, V.; Nogueira, R. P.; Bolfarini, C., Corrosion resistance of Fe-based amorphous alloys. *Journal of Alloys and Compounds* **2014,** 586, S105-S110.
2. Han, Y.; Chang, C. T.; Zhu, S. L.; Inoue, A.; Louzguine-Luzgin, D. V.; Shalaan, E.; Al-Marzouki, F., Fe-based soft magnetic amorphous alloys with high saturation magnetization above 1.5 T and high corrosion resistance. *Intermetallics* **2014,** 54, 169-175.
3. Han, Y.; Kong, F. L.; Han, F. F.; Inoue, A.; Zhu, S. L.; Shalaan, E.; Al-Marzouki, F., New Fe-based soft magnetic amorphous alloys with high saturation magnetization and good corrosion resistance for dust core application. *Intermetallics* **2016,** 76, 18-25.
4. Fan, Y.; Zhang, S.; Xu, X.; Miao, J.; Zhang, W.; Wang, T.; Chen, C.; Wei, R.; Li, F., Effect of the substitution of Si for B on thermal stability, magnetic properties and corrosion resistance in novel Fe-rich amorphous soft magnetic alloy. *Intermetallics* **2021,** 138, 107306.
5. Vasić, M. M.; Simatović, I. S.; Radović, L.; Minić, D. M., Influence of microstructure of composite amorphous/nanocrystalline Fe72Ni8Si10B10 alloy on the corrosion behavior in various environments. *Corrosion Science* **2022,** 204, 110403.
6. Berger, J. E.; Jorge, A. M.; Koga, G. Y.; Roche, V.; Kiminami, C. S.; Bolfarini, C.; Botta, W. J., Influence of chromium concentration and partial crystallization on the corrosion resistance of FeCrNiB amorphous alloys. *Materials Characterization* **2021,** 179, 111369.
7. Wu, L.; Zhou, Z.; Zhang, K.; Zhang, X.; Wang, G., Electrochemical and passive film evaluation on the corrosion resistance variation of Fe-based amorphous coating affected by high temperature. *Journal of Non-Crystalline Solids* **2022,** 597, 121892.
8. Coimbrão, D. D.; Zepon, G.; Koga, G. Y.; Godoy Pérez, D. A.; Paes de Almeida, F. H.; Roche, V.; Lepretre, J. C.; Jorge, A. M.; Kiminami, C. S.; Bolfarini, C.; Inoue, A.; Botta, W. J., Corrosion properties of amorphous, partially, and fully crystallized Fe68Cr8Mo4Nb4B16 alloy. *Journal of Alloys and Compounds* **2020,** 826, 154123.
9. Koga, G. Y.; Nogueira, R. P.; Roche, V.; Yavari, A. R.; Melle, A. K.; Gallego, J.; Bolfarini, C.; Kiminami, C. S.; Botta, W. J., Corrosion properties of Fe–Cr–Nb–B amorphous alloys and coatings. *Surface and Coatings Technology* **2014,** 254, 238-243.
10. Liu, Y.; Li, J.; Sun, Y.; He, A.; Dong, Y.; Wang, Y., Effect of annealing temperature on magnetic properties and corrosion resistance of Fe75.8Si12B8Nb2.6Cu0.6P1 alloy. *Journal of Materials Research and Technology* **2021,** 15, 3880-3894.
11. Sunbul, S. E.; Akyol, S.; Onal, S.; Ozturk, S.; Sozeri, H.; Icin, K., Effect of Co, Cu, and Mo alloying metals on electrochemical and magnetic properties of Fe-B alloy. *Journal of Alloys and Compounds* **2023,** 947, 169652.
12. Meghwal, A.; Pinches, S.; King, H. J.; Schulz, C.; Stanford, N.; Hall, C.; Berndt, C. C.; Ang, A. S. M., Fe-based amorphous coating for high-temperature wear, marine and low pH environments. *Materialia* **2022,** 25, 101549.
